# Sustained effect of couples' HIV counselling and testing on risk reduction among Zambian HIV serodiscordant couples

**DOI:** 10.1136/sextrans-2016-052743

**Published:** 2017-01-12

**Authors:** Kristin M Wall, William Kilembe, Bellington Vwalika, Lisa B Haddad, Shabir Lakhi, Udodirim Onwubiko, Naw Htee Khu, Ilene Brill, Roy Chavuma, Cheswa Vwalika, Lawrence Mwananyanda, Elwyn Chomba, Joseph Mulenga, Amanda Tichacek, Susan Allen

**Affiliations:** 1 Rwanda Zambia HIV Research Group, Department of Pathology and Laboratory Medicine, School of Medicine and Hubert Department of Global Health, Rollins School of Public Health, Emory University, Atlanta, Georgia, USA; 2 Department of Epidemiology, Rollins School of Public Health, Laney Graduate School, Emory University, Atlanta, Georgia, USA; 3 Department of Gynaecology and Obstetrics (BV), Internal Medicine (SL) and Surgery (RC), School of Medicine, University of Zambia, Lusaka, Zambia; 4 Department of Gynaecology and Obstetrics, Emory University, School of Medicine, Atlanta, Georgia, USA; 5 Department of Epidemiology, Ryals School of Public Health, University of Alabama at Birmingham, Birmingham, Alabama, USA; 6 Ministry of Community Development, Mother and Child Health, Lusaka, Zambia

**Keywords:** AFRICA, AIDS, BEHAVIOURAL INTERVENTIONS, EPIDEMIOLOGY (GENERAL), SEXUAL BEHAVIOUR

## Abstract

**Background:**

We present temporal trends in self-reported and biological markers of unprotected sex and sex with concurrent partners in discordant couples receiving couples' voluntary HIV counselling and testing (CVCT).

**Methods:**

Heterosexual Zambian HIV-serodiscordant couples were enrolled into longitudinal follow-up in an open cohort (1994–2012). Multivariable Anderson-Gill models explored predictors of self-report and biological indicators of unprotected sex within (including sperm on a vaginal swab, incident pregnancy or incident linked HIV infection) and outside (including self-report, STI and unlinked HIV infection) the union. Measures of secular trends in baseline measures were also examined.

**Results:**

At enrolment of 3049 couples, men were 35 years old on average, women were 29 years, and couples had been together for an average of 7 years. *M+F− couples* reported an average of 16.6 unprotected sex acts in the 3 months prior to enrolment (pre-CVCT), dropping to 5.3 in the >0–3 month interval, and 2.0 in >6 month intervals (p-trend <0.001). Corresponding values for *M−F+ couples* were 22.4 unprotected sex acts in the 3 months prior enrolment, dropping to 5.2 in the >0–3 month interval, and 3.1 in >6 month intervals (p-trend <0.001). Significant reductions in self-report and biological markers of outside partners were also noted. Predictors of unprotected sex between study partners after CVCT included prevalent pregnancy (adjusted HR, aHR=1.6–1.9); HIV+ men being circumcised (aHR=1.2); and HIV− women reporting sex with outside partners (aHR=1.3), alcohol (aHR=1.2), injectable (aHR=1.4) or oral (aHR=1.4) contraception use. Fertility intentions were also predictive of unprotected sex (aHR=1.2–1.4). Secular trends indicated steady declines in reported outside partners and STIs.

**Conclusion:**

Reductions in self-reported unprotected sex after CVCT were substantial and sustained. Reinforced risk-reduction counselling in pregnant couples, couples desiring children and couples with HIV− women having outside partners or using alcohol or injectable or oral contraception are indicated.

## Introduction

Roughly half of couples affected by HIV in sub-Saharan Africa are in HIV-serodiscordant relationships (one partner HIV-positive, one partner HIV-negative),^1^ and the majority of new infections originate with the HIV-positive index rather than concurrent, outside partners.^2^ Couples' voluntary HIV counselling and testing (CVCT) has been associated with significant reductions in HIV incidence in serodiscordant couples as well as STIs and unplanned pregnancies.^3–12^

These benefits are, in large part, the result of a decrease in unprotected sexual exposures. In a prospective study in Rwanda, where CVCT is now standard of care, condom use among serodiscordant couples increased from 4% at enrolment to 57% after 1 year of post-CVCT follow-up, and condom use was associated with lower HIV seroconversion rates.^5^ Among mixed serostatus couples, women in couples with at least one HIV-positive partner reported decreased frequency of coercive sex after joint counselling.^13^ In Zambia, where CVCT programmes were implemented began 20 years ago, a study among serodiscordant couples showed that self-reported condom use increased from 3% of sexual acts prior to CVCT to >80% after 1 year of follow-up, though under-reporting of unprotected sex was common.^11^

The primary objective of our analysis was to assess changes in indicators of unprotected sex with the study partner measured at enrolment (reflecting time prior to CVCT services) and over time post-CVCT enrolment among HIV-serodiscordant heterosexual couples (M+F−, M−F+). We compare self-report and biological markers of unprotected sex to better quantify under-reporting. Finally, important secular trends in self-report and biological variables from 1994 to 2012 are described.

## Methods

### Ethics

This study was approved by the University of Zambia (Research Ethics Committee IRB00001131) and the Emory Institutional Review Board (IRB00000453). Joint written informed consent was obtained from all participating couples.

### Study design, participants and data collection

We analysed data from heterosexual HIV-serodiscordant couples recruited from CVCT services and enrolled in a longitudinal open cohort study of HIV transmission in Lusaka, Zambia between 1994 and 2012. CVCT services include group counselling, rapid HIV testing and post-test couples' counselling with mutual disclosure of results.^14^ ^15^ Guidelines for CVCT as prevention are available from WHO (http://www.who.int/hiv/pub/guidelines/9789241501972/en/) and couples' HIV counsellor training guidelines developed with participation of our research team are available from the Centers for Disease Control (http://www.cdc.gov/globalaids/resources/prevention/chct.html). Enrolled couples were followed every 3 months at the research clinic and received meals, childcare and transport reimbursement for each visit. Couples were censored at HIV seroconversion of the negative partner or couple separation, relocation, voluntary withdrawal or death. When antiretroviral treatment (ART) was available in government clinics beginning in 2007, HIV-positive partners were referred for triage; if and when they initiated ART they were released from the cohort. Due to funding limitations, follow-up was truncated at 24 months beginning in 2010 and 12 months beginning in 2011.

Baseline demographical, behavioural and clinical data were collected at enrolment.^16^ This included age, duration of cohabitation, number of prior pregnancies, literacy, alcohol use, fertility intentions, history of STI and clinical stage and viral load of the HIV+ partner. Time-varying covariates including contraception method, self-reported sex with and without a condom with study partner (recorded on coital logs provided by the research project), pregnancy status, self-reported sex with outside partners, sperm on a vaginal swab wet mount, STI diagnosed clinically, laboratory diagnosis of trichomonas in the woman (vaginal swab wet mount) and syphilis in either partner (rapid plasma regain (RPR) serology with later Treponema pallidum haemagglutination assay (TPHA) confirmation^17^) were collected at baseline and follow-up visits.

### Outcomes of interest: unprotected sex with the study partner and sex with outside partners

Time-varying composite indicators of unprotected sex with the study partner and sex with outside partners were created including self-reported and biological markers. Quarterly intervals with unprotected sex with study partner were defined as one or more of: couple-reported condomless sex with study partner in the previous 3 months, incident pregnancy, incident seroconversion of the HIV-negative partner that was genetically linked to the positive partner, and sperm presence on a vaginal swab wet mount. The corresponding composite variable for sex with outside partners included self-report of outside partners and/or incident syphilis, trichomonas or unlinked HIV infection.

### Changes in unprotected sex with the study partner and sex with outside partners before and after CVCT and over follow-up

The prevalence of individual indicators at baseline and during follow-up and the prevalence of the composite variables during follow-up is presented graphically. Composite variables could not be used at the enrolment visit for several reasons: first, couples had been tested and counselled a mean of 7 days before enrolment and had begun using condoms immediately, thus sperm (which is only detectable in vaginal swabs for a few days after intercourse) did not reflect ‘pre-CVCT’ behaviours; second, prevalent pregnancy at baseline was not comparable to incident pregnancy thereafter; finally, trichomonas and positive RPR serology for syphilis could reflect disease acquired more than 3 months prior to enrolment and thus would not be comparable to incidence during the post-enrolment quarterly follow-up intervals.

Thus, only self-report of outside partners is compared between enrolment and follow-up, whereas tests of trend were performed to assess effects in the composite variable over time after enrolment. In a sub-analysis, we examined the magnitude of under-reporting of unprotected sex, overall and over time, by comparing self-report with biological indicators.

### Predictors of unprotected sex with the study partner

Couple demographics and exposures of interest were described (counts and percentages for categorical variables, means and SDs for continuous variables, tabulated over all study intervals) by whether or not the couple was positive for the time-varying composite of unprotected sex within the couple. Unadjusted associations between covariates and the outcome were calculated via survival analysis accommodating both fixed and time-varying covariates (Anderson-Gill models). Factors significant in crude analyses were entered into multivariable models after assessing for multi-collinearity. We also built models adjusting for fertility intentions, which were collected from 2002 to 2006. To explore the possibility of informative censoring over long follow-up times, models were also run using inverse probability of censoring weighting (IPCW). To explore the possibility of important differences between the composite indicator and self-reported unprotected sex, models were additionally run using only self-reported unprotected sex as the outcome.

### Self-reported and biological indicators of unprotected sex by calendar time of enrolment

Finally, we tabulated self-reported and biological indicators of unprotected sex over calendar time of enrolment to explore secular trends. Proportions and 95% CIs are shown. A test of trend was performed to assess secular trends.

Data were analysed using SAS V.9.4 (SAS Institute, Inc, Cary, North Carolina, USA).

## Results

### Changes in unprotected sex with the study partner before and after CVCT and over follow-up

**Figure 1:**
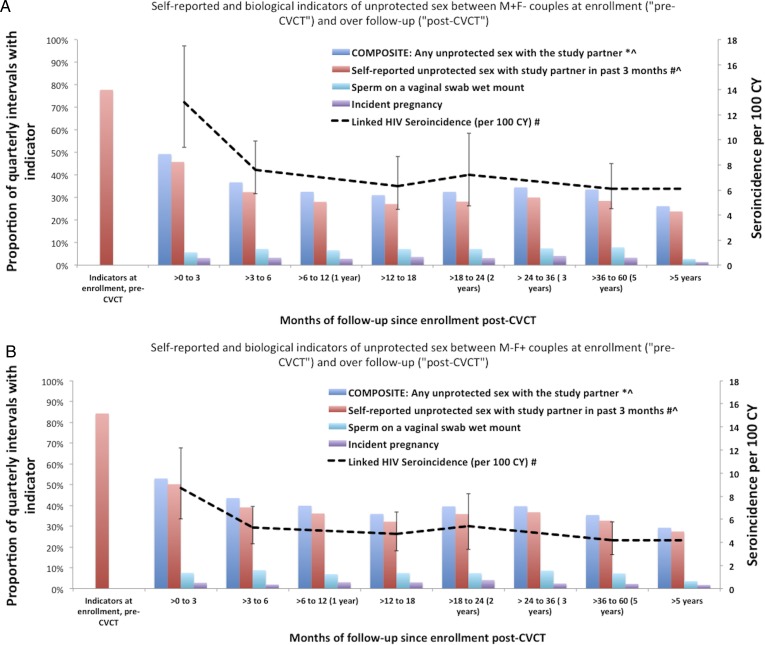
Self-reported and biological indicators of unprotected sex between HIV-serodiscordant couples at enrolment (‘pre-couples’ voluntary HIV counselling and testing, CVCT’) and over follow-up (‘post-CVCT’) (A: M+F− couples; B: M−F+ couples). *Composite of: any self-reported unprotected sex with the study partner in the past 3 months, incident pregnancy (only during follow-up), sperm present on a vaginal swab wet mount and incident linked HIV seroconversion. Linked HIV seroincidence per 100 CY refer to z-axis. ^#^p<0.01 for differences between pre-CVCT versus first follow-up. ^^^p<0.01 for downward trend over post-CVCT follow-up time. Proportions are calculated among all couples; linked HIV seroincidence is calculated excluding couples who experienced an unlinked infection. CVCT, couples' HIV voluntary counselling and testing; CY, couple-years.

Among 3049 couples (N=1393 M+F−, N=1656 M−F+), 57% had at least 1 year of follow-up, 35% had ≥2 years, and 22% had ≥3 years (average follow-up of 1.5 years per couple) (figure 1A, B).

*M+F− couples* reported an average of 16.6 unprotected sex acts within the past 3 months at enrolment (SD=22.4), dropping to 5.3 in the >0–3 month interval (SD=11.9), and 2.0 in >6 month intervals (SD=7.8) (p-trend<0.001). *M−F+ couples* reported an average of 22.4 unprotected sex acts within the past 3 months at enrolment (SD=25.2), dropping to 5.2 in the >0–3 month interval (SD=12.3) and 3.1 in >6 month intervals (SD=11.1) (p-trend <0.001).

Reporting at least one unprotected sexual exposure per quarter decreased significantly from enrolment (reflecting sexual behaviour prior to CVCT) to the >0–3 month interval immediately after CVCT (78–46% of intervals for M+F− and 84–50% for M−F+ couples, p<0.01). Self-reported unprotected sex decreased thereafter to an average of 28% of intervals for M+F− couples and 43% for M−F+ couples over the remainder of follow-up visits (>3 months, p<0.01).

Linked HIV seroconversion rates were highest in the first month >0–3 follow-up interval, reflecting some infections acquired prior to CVCT; the incidence declined significantly between the first and later follow-up intervals (p<0.01), whereas rates after the first quarterly interval remained stable. Similarly, self-reported unprotected sex decreased significantly from pre-CVCT to first quarterly follow-up and had a significant subsequent downward trend (p<0.01). Sperm presence on a vaginal swab wet mount and incident pregnancy did not change significantly during follow-up. These findings were similar for M+F− and M−F+ couples (figure 1A, B).

In the sub-analysis exploring the potential for under-reporting of unprotected sex during follow-up overall, 32% of incident pregnancies, 58% of linked seroconversions and 45% of sperm-positive wet smears occurred during intervals in which M+F− couples reported no unprotected sex. For M−F+ couples, 29% of pregnancies, 39% of linked seroconversions and 42% of sperm-positive wet smears occurred during intervals in which couples reported no unprotected sex. Over time, we did not observe significant changes in the amount of misclassification.

### Predictors of unprotected sex with the study partner

Cohort demographics are shown in table 1. For M+F− couples, predictors of the composite indicator of unprotected sex with the study partner after CVCT included: use of injectable (adjusted HR, aHR=1.4; 95% CI 1.3 to 1.6) or oral contraceptive pill (OCP, aHR=1.4; 95% CI 1.2 to 1.5) versus condoms only; being currently pregnant (aHR=1.9; 95% CI 1.7 to 2.0) or post-partum (aHR=0.8; 95% CI 0.7 to 0.99) versus not pregnant; woman's baseline alcohol use (aHR=1.2; 95% CI 1.0 to 1.3) versus non-use; the man being circumcised (aHR=1.2; 95% CI 1.0 to 1.5); and the woman reporting outside partners (aHR=1.3; 95% CI 1.1 to 1.6) controlling for age, men's self-reported outside sex, and follow-up time since enrolment (>0–3 months vs later) (table 1). Later follow-up intervals were protectively compared with the first (>0–3 month) follow-up interval (p<0.0001, not shown in table). In a model additionally controlling for the woman's fertility intentions, findings remained similar and desire for more children was predictive of unprotected sex (aHR=1.4).

**Table 1:** Factors associated with the composite indicator of unprotected sex* between M+F− and M−F+ couples, univariable and multivariable analyses

	M+F− couples								M−F+ couples							
						Multivariate Model†								Multivariate Model†		
	Total cohort (N interval, % or mean, SD)		Any unprotected sex (N interval, % or mean, SD)		Unadjusted p Value (2-tail)	aHR	95% CI	p Value (two-tail)	Total cohort (N interval, % or mean, SD)		Any unprotected sex (N interval, % or mean, SD)		Unadjusted p value (two-tail)	aHR	95% CI	p Value (two-tail)
Demographics																
Man age (per year increase)‡	35.2	7.6	34.0	7.4	<0.0001	0.98	0.98 to 0.99	<0.0001	35.2	8.6	34.3	8.3	<0.0001	0.99	0.98 to 0.99	<0.0001
Woman age (per year increase) ‡	28.4	6.8	27.4	6.6	<0.0001				28.6	6.7	27.9	6.5	<0.0001			
Years cohabiting (per year increase) ‡	8.1	6.5	7.3	6.0	<0.0001				6.1	5.8	5.6	5.4	<0.001			
Woman alcohol use last year																
Yes	2549	17%	1057	18%	0.022	1.15	1.01 to 1.31	0.033	3606	24%	1550	23%	0.729			
No	12 582	83%	4727	82%		Ref			11 585	76%	5119	77%				
Man alcohol use last year																
Yes	10 152	67%	3964	68%	0.033	1.11	0.99 to 1.24	0.088	11 181	73%	4979	74%	0.166			
No	5034	33%	1825	32%		Ref			4034	27%	1722	26%				
Family planning and sexual behaviour																
Contraceptive method (time-varying)																
Condoms alone	9553	62%	3701	63%		Ref			10 318	67%	4698	69%				
OCPs	2128	14%	969	16%	<0.0001	1.37	1.22 to 1.54	<0.0001	2061	13%	910	13%	0.368			
Injectables	2011	13%	794	13%	0.002	1.43	1.25 to 1.64	<0.0001	1975	13%	808	12%	0.271			
Permanent method, IUD, implant	1711	11%	437	7%	0.508	1.14	0.90 to 1.44	0.293	1157	7%	405	6%	0.716			
Pregnancy status (time-varying)																
Not pregnant	11 922	87%	4427	80%		Ref			12 167	89%	5315	85%		Ref		
Pregnant (not incident)	1346	10%	938	17%	<0.0001	1.85	1.71 to 2.01	<0.0001	1140	8%	857	14%	<0.0001	1.56	1.46 to 1.66	<0.0001
Post-partum (up to 6 months)	510	4%	136	2%	0.004	0.83	0.70 to 0.99	0.038	341	2%	108	2%	0.005	0.81	0.68 to 0.95	0.0107
Number of previous pregnancies (per pregnancy increase)‡	3.7	2.5	3.5	2.3	0.001				3.4	2.3	3.3	2.1	0.032			
Fertility intentions of woman																
Yes, next year	1051	18%	418	21%	0.045				1642	23%	693	24%	0.372			
Yes, but not next year	1217	21%	472	24%	0.005				1770	25%	772	27%	0.063			
Do not know/no	3610	61%	1076	55%					3750	52%	1435	49%				
Fertility intentions of man																
Yes, next year	671	13%	259	15%	0.126				1145	18%	511	20%	0.023			
Yes, but not next year	1652	32%	573	34%	0.160				2556	39%	1073	42%	0.016			
Do not know/No	2880	55%	869	51%					2829	43%	994	39%				
Woman self-reporting sex with outside partner (time-varying)																
Yes	205	1%	115	2%	<0.001	1.30	1.09 to 1.55	0.003	236	2%	131	2%	0.241			
No	14 848	99%	5720	98%		Ref			14 150	98%	6408	98%				
Clinical																
Circumcised male partner																
Yes	1508	10%	619	11%	0.05	1.23	1.03 to 1.46	0.021	2587	17%	1084	16%	0.249			
No	13 876	90%	5266	89%		Ref			12 963	83%	5745	84%				

Fertility intentions collected from 2002 to 2011; viral load collected from 1999.

p Values are two-tailed.

*Composite of: any self-reported unprotected sex with the study partner in the past 3 months, incident pregnancy, sperm present on a vaginal swab wet prep and incident linked HIV seroconversion.

Log viral load of positive partner (per log10 copies/mL increase), Nyanja literacy and man self-reporting sex with outside partner were not associated (p>0.05).

†Controlling for time since enrolment (>0–3 months vs later, p<0.005).

‡Indicates a continuous variable, mean and standard deviation reported.

Nyanja: Commonly spoken language in Lusaka, Zambia.

Stage of HIV, income and man’s literacy were not significant.

IUD, copper intrauterine device; OCP, oral contraceptive pill.

For M−F+ couples, predictors of the composite indicator of unprotected sex with the study partner after CVCT included being currently pregnant (aHR=1.6; 95% CI 1.5 to 1.7) or post-partum (aHR=0.8; 95% CI 0.7 to 0.95) versus not pregnant/post-partum, controlling for age and increasing follow-up time since enrolment (table 1). Increasing follow-up interval (>0–3 months vs later) since enrolment was again protective for unprotected sex (p<0.0001, not shown in table). In a model additionally controlling for the man's fertility intentions, findings remained similar and desire for more children was predictive of unprotected sex (aHR=1.2).

Models using IPCW methods to explore the potential influence of selective loss to follow-up produced very similar findings with the exception of post-partum status for M+F− couples, which became non-significant. Models using the self-reported unprotected sex as the outcome showed similar results, with the exception that women's self-reported outside sex and post-partum periods were not significant.

### Changes in indicators of sex with outside partners over follow-up time

**Figure 2:**
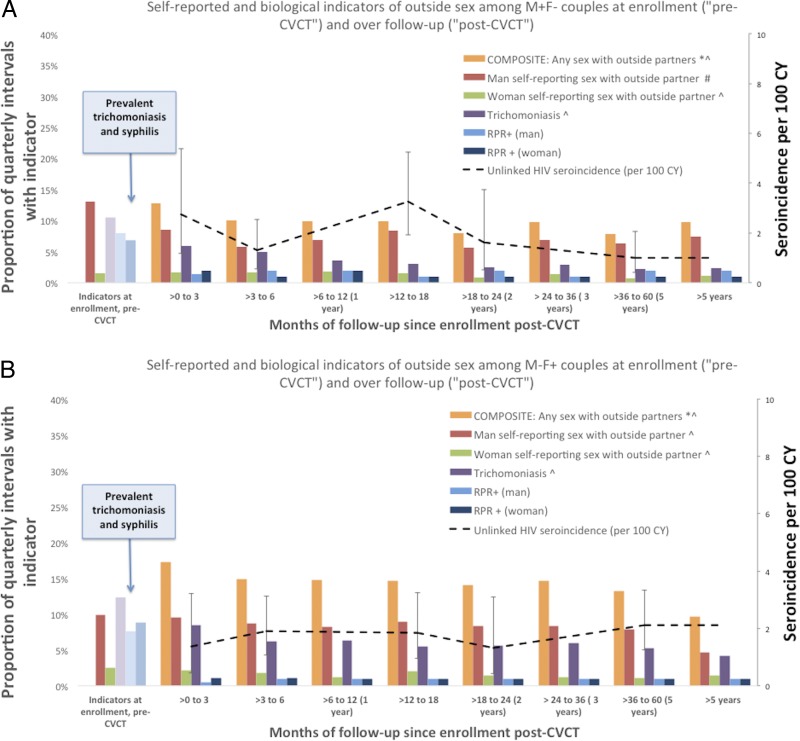
Self-reported and biological indicators of outside sex among HIV-serodiscordant couples at enrolment (‘pre-couples’ voluntary HIV counselling and testing, CVCT’) and over follow-up (‘post-CVCT’) (A: M+F− couples; B: M−F+ couples).*Composite of: any self-reported sex with outside partners in the past 3 months for men or women, incident unlinked HIV seroconversion incident trichomoniasis, and incident syphilis (prevalent trichomoniasis and prevalent syphilis not included in composite indicator). Unlinked HIV seroincidence per 100 CY refer to z-axis. ^#^p<0.01 for differences between pre-CVCT versus first follow-up. ^^^p<0.01 for downward trend over post-CVCT follow-up time. CY, couple-years; CVCT, couples' HIV voluntary counselling and testing; RPR, rapid plasma regain.

HIV+ men were more likely to report sex with outside partners in the 3 months prior to enrolment than HIV− men (14% vs 10%, p<0.01) (figure 2A, B). Self-reported outside partners among HIV+ men decreased significantly by half immediately after CVCT, with no significant trend over subsequent follow-up. Self-reported outside partners among HIV− men showed no change pre- versus post-CVCT, but did have a significant overall downward trend during post-CVCT follow-up.

Few women ever reported outside partners, and there was a significant downward trend during follow-up. The incidence of trichomonas also decreased significantly over follow-up, as did the composite variable.

### Self-reported and biological indicators of unprotected sex by calendar time of enrolment

**Figure 3:**
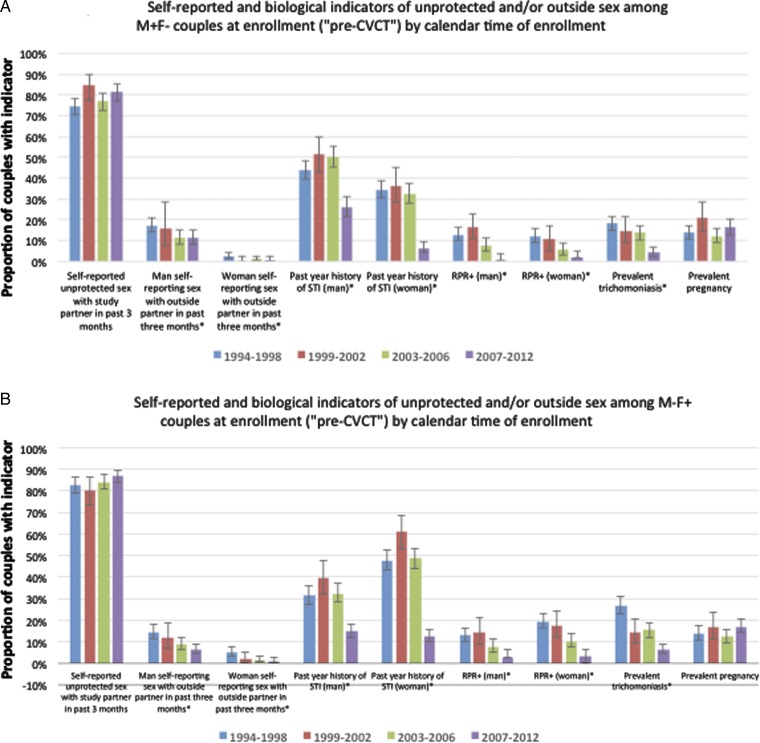
Self-reported and biological indicators of unprotected among HIV-serodiscordant couples at enrolment (‘pre-couples’ voluntary HIV counselling and testing, CVCT’) and over follow-up (‘post-CVCT’) (A: M+F− couples; B: M−F+ couples). *p-trend <0.01 for differences over calendar time. 95% error bars (Clopper-Pearson exact) shown. CVCT, couples' HIV voluntary counselling and testing; RPR, rapid plasma regain.

Self-reported unprotected sex with the study partner at enrolment and prevalent pregnancy at enrolment did not significantly change over calendar time between 1994 and 2012 (figure 3A, B). In contrast, there were substantial declines (p-trend <0.01) in history of STI in the year prior to enrolment: comparing the 1994–1998 to the 2007–2012 time frames, history of STI decreased from 44% to 26% among HIV+ men, 35% to 7% among HIV− women, 31% to 15% among HIV− men and 48% to 13% among HIV+ women. Interestingly, history of STI peaked in the 1999–2002 time frame. There were also marked declines in men reporting sex with outside partners, noticeably beginning in 2003–2006. HIV+ women had a higher prevalence of trichomonas than HIV− women in 1994–1998 (27% vs 18%, p<0.05), with both showing a decline in 2007–2012 (4% of HIV+ and 6% of HIV). Positive RPR serology was highest in HIV+ women at all time points, with a high of 19% in the 1994–1998 time frame, with a significant over time in all groups.

## Discussion

This study examined the impact of CVCT on sexual risk behaviour over time in a large cohort of HIV-serodiscordant heterosexual couples in Zambia. As expected, there were significant reductions in self-reported measures of unprotected sex with the study partner after CVCT with no evidence of relapse in these risk behaviours with time. Similarly, measures of concurrent partnerships showed decreases after CVCT. Predictors of residual unprotected sex within marriage highlight opportunities for targeted counselling. These findings support WHO guidelines to provide joint HIV testing and counselling for prevention.

A strength of this analysis is the use of the composite indicator to correct for known outcome misclassification and quantify under-reporting: 29–32% of incident pregnancies, 39–58% of linked seroconversions and 42–45% of sperm-positive vaginal smears occurred during intervals in which couples reported no unprotected sex. This implies that the actual number of unprotected acts is actually up to almost 60% higher than the self-reported numbers. In addition, multivariate models using the composite outcome measure detected two additional significant predictors: a protective effect during the post-partum period for all couples and increased risk of exposure within marriage if the HIV− woman also reported outside partners.

Our finding that OCP and injectable use was significantly associated with the composite measure of unprotected sex among M+F− couples, which confirmed our previous findings that bivariate associations between OCPs/injectables and HIV in women were eliminated in analyses controlling for measures of unprotected sex.^18^ ^19^ The increase in risky sexual behaviour in this sub-group may explain why other studies that have not controlled for confounding by unprotected sex have seen increased rates of seroconversion for oral and injectable contraceptive users.^20^ Reinforced dual-method use counselling for women using OCPs and injectable hormonal contraception is warranted.

Pregnancy was associated with unprotected sex in both M+F− and M−F+ couples. This might be explained by misconceptions, such as traditional beliefs that semen nourishes the fetus^21^ ^22^ or simply that couples engaging in unprotected sex are more likely to get pregnant and continue this behaviour. Studies to understand why discordant couples engage in unprotected sex during pregnancy are warranted, along with reinforced risk-reduction counselling in pregnant HIV-serodiscordant couples.

The use of alcohol by women in M+F− couples was predictive of the composite indicator of unprotected sex, as was HIV− women reporting concurrent sexual partners. Though uncommon, this may be an important risk profile to study. Additionally, the association of circumcision and increased unprotected sex among M+F− couples may be the result of conflicting messages about protection from HIV (only HIV− men) versus other STI (both HIV+ and HIV− men).^23^ Additional counselling in M+F− couples is needed, along with further research to explore this finding.

Ndase *et al*^24^ found significant increases in the proportion of uninfected partners within serodiscordant couples self-reporting sex with outside partners after 2 years of follow-up in seven African countries. In contrast, our study showed either a decrease or no change in self-reported outside partners, but a substantial decline in syphilis and trichomonas. This difference might be due to our use of biological markers and/or could be a reflection of the relationship stability of couples residing in a region with relatively homogenous strong religious and cultural beliefs.

Secular trends in baseline measures indicate that the environment we were working in was changing with steady reductions in self-reported STI and laboratory diagnosis of syphilis and trichomonas beginning in 2002 and most notable after 2007. Although this coincided with declines in reported outside partners, there may also have been an important contribution from HIV/STI prevention messages and/or STI treatment programme scale-up outside our study.

These findings should be interpreted in light of several considerations. As detailed, self-report of sexual behaviours is subject to under-reporting (though some ‘misclassification’ could be due to condom failure); meanwhile, biological markers of unprotected sex are insensitive. Given high levels of misclassification, we feel that evaluating multiple indicators of unprotected sex is critical and supports our rationale for modelling the composite indicator. Though we corrected for instances of under-reporting when possible using biological measures, those measures are themselves imperfect, possibly leading to residual misclassification. However, since we did not observe that misclassification changed over time, the overall trends and relative effects observed should not be biased. Under-reporting of unprotected sex is common and mitigating this bias should be a major part of study design. Strategies include sexual diaries to enhance recall,^25^ and Audio Computer-Assisted Self-Interviewing (ACASI) which may increase reporting of sensitive high-risk behaviours.^26^ A recent study using biomarkers to compare ACASI to interview in Zimbabwe confirmed under-reporting in both groups, reinforcing the usefulness of biological markers.^27^

In addition, the generalisability of our findings must be interpreted in light of a self-selection process that may lead to more health-conscious couples being retained for longer. Selective loss to follow-up, extensively evaluated and reported previously in our cohorts^28^ implies that our findings are more generalisable to older couples who live closer to the clinic in which the female partner had an older age of first sexual intercourse and, in M−F+ couples, generalisable to couples with increased income. IPCW findings did not indicate that informative censoring was biasing our results. Finally, not all indicators of unprotected sex (eg, sperm presence on a vaginal swab wet prep and incident pregnancy) could definitively be attributed to the study partner, though we assume that the majority of instances of these indicators, which can only be measured in women, are related to unprotected sex with the study partner given the low incidence of genetically unlinked infections and that women report few outside contacts.

## Conclusion

Our findings indicate that in HIV-serodiscordant heterosexual couples, reductions in unprotected sex and outside sex after CVCT are significant and sustained. Condom use was imperfect and associated with specific characteristics that should elicit additional counselling. When possible, biological markers should be assessed along with self-report.

**Key messages**

- Couples' voluntary HIV counselling and testing (CVCT) intervention reduces HIV/STI incidence in HIV-serodiscordant couples by reducing unprotected sex.
- Reductions in unprotected sex and indicators of outside sex after CVCT are significant and sustained over long-term follow-up in HIV-serodiscordant heterosexual couples.
- Almost 40% of couples continued to have some indicator of unprotected sex and targeted risk reduction counselling is warranted.
- Couples' voluntary HIV counselling and testing should be scaled up per WHO guidelines.
